# Hereditary angioedema without deficiency of C1 inhibitor: response to therapy

**DOI:** 10.1186/1939-4551-8-S1-A130

**Published:** 2015-04-08

**Authors:** Fabiane Milena Castro Araújo Pimenta, Vivian Alves Costa, Ana Karolinne Burlamaqui Melo, Aline Lury Aoki, Sandra Mitie Palma, Rosemeire Navickas Constantino-Silva, Neusa Falbo Wandalsen, Anete Grumach

**Affiliations:** 1 Faculty of Medicine ABC, SP, Brazil

## Background

Hereditary angioedema (HAE) with normal C1 esterase inhibitor (C1INH) was described for the first time in 2000. It was characterized by subcutaneous, gastrointestinal and laryngeal edema with familial history. Triggering factors are: stress, hormonal factors, trauma and infections. The authors evaluate response to therapy in patients with HAE without C1-INH deficiency.

## Methods

It was analized therapeutical response to hereditary angioedema without deficiency of C1INH. Patients with clinical symptoms compatible with HAE have been included after normal quantitative and functional C1INH levels and positive family history for HAE, independent of factor XII mutation.

## Results

Nineteen patients have been identified (2M:17F; 20-60 years old). The following therapies were oriented: combined contraceptive substitution for progestagen (10/19); treatment with progestagen (2/19); tranexamic acid (15/19): 1250mg (2), 1000mg (1), 750 (5), 500 mg (4), 250 mg (1); oxandrolon (5/19) (0.5 mg-5mg/day), danazol 200mg/day (1/19) and combined therapy woth oxandrolon and tranexamic acid in two patients. Icatibant was used in seven patients with clinical improvement. One of them reported increasing frequency of attacks after repeated use of this drug. Two patients received fresh frozen plasma during attacks with clinical improvement.

## Conclusions

HAE without C1-INH deficiency has no established treatment. Clinical improvement was evident with the exclusion of combined contraceptives. The majority of the patients presented clinical response to tranexamic acid in variable doses. Icatibant was adequate for the therapy of attacks.

